# An Anisotropic Model for Magnetostriction and Magnetization Computing for Noise Generation in Electric Devices

**DOI:** 10.3390/s16040553

**Published:** 2016-04-16

**Authors:** Serigne Saliou Mbengue, Nicolas Buiron, Vincent Lanfranchi

**Affiliations:** 1 Sorbonne universités, Université de Technologie de Compiègne (UTC) Laboratoire Roberval (CNRS UMR7337), 60203 Compiègne Cedex, France; nicolas.buiron@utc.fr; 2 Sorbonne universités, UTC Laboratoire Electromécanique de Compiègne (EA1006), 60203 Compiègne Cedex, France; vincent.lanfranchi@utc.fr

**Keywords:** anhysteretic, magnetostriction, magnetization, anisotropy, transformer, finite elements, vibration, noise

## Abstract

During the manufacturing process and use of ferromagnetic sheets, operations such as rolling, cutting, and tightening induce anisotropy that changes the material’s behavior. Consequently for more accuracy in magnetization and magnetostriction calculations in electric devices such as transformers, anisotropic effects should be considered. In the following sections, we give an overview of a macroscopic model which takes into account the magnetic and magnetoelastic anisotropy of the material for both magnetization and magnetostriction computing. Firstly, a comparison between the model results and measurements from a Single Sheet Tester (SST) and values will be shown. Secondly, the model is integrated in a finite elements code to predict magnetostrictive deformation of an in-house test bench which is a stack of 40 sheets glued together by the Vacuum-Pressure Impregnation (VPI) method. Measurements on the test bench and Finite Elements results are presented.

## 1. Introduction

Because of energy harvesting systems, magnetostrictive actuator design and noise reduction issues in electric devices, several papers deal with the topic of magnetostriction of ferromagnetic materials. In fact, giant magnetostrictive materials (GMMs) are widely used in transduction applications. However the magnetostriction of ferromagnetic sheets used in electric devices such as transformers is an important source of noise [1]. Despite the low magnetostriction of those ferromagnetic sheets (on the order of some µm/m for silicon iron alloy), the magnetostriction effects can be amplified by structure resonance thus resulting in loud noise radiation.

The complexity of the phenomenon leads to several more or less accurate models depending on the application. In this context, relatively recent works [2,3,4,5] dealing with structure magnetostriction computation or measurement show that the magnetic flux line direction change significantly affects results. In other word, magnetostriction of ferromagnetic materials is highly anisotropic. As shown in [6] it is important to take this anisotropic effect into account in applications involving rotational magnetic flux density. Therefore a bidirectional anisotropic model is proposed in [7]. In this paper, the authors use an elliptical function to modulate the magnitude of the magnetostriction according to the magnetization direction. Then magnetostriction along the rolling (RD) and the transverse (TD) directions are different. However, the magnetostriction in other directions is not shown. In [8,9], the anisotropic effect on magnetostriction is modeled thanks to the analogy between magnetostriction and viscoelastic behavior. Two equivalent Poisson ratios are identified from measurements to change the value of the magnetostriction magnitude. Like the previous approach [7], the magnetostriction measured along RD and TD are shown. The model is also validated in these two directions.

In this paper, a multidirectional anisotropic model calculating both magnetization and magnetostriction in ferromagnetic steel sheets is shown. This approach enables one to take into account both magnetic and magnetostrictive anisotropy in the same model. Therefore the accuracy of the simulation results can be improved. The model is based on the minimization of the macroscopic total energy which depends on different energy terms written in a macroscopic scale as a function of magnetization and some parameters. The assumptions and methods for identifying these parameters from experimental results will be detailed in the following sections. Then a comparison between measurements on a single sheet tester and simulations will be presented. Then the model is used in a finite elements code for predicting the magnetostriction of an in-house test bench which is a stack of 40 sheets glued by the Vacuum-Pressure Impregnation (VPI) method. A comparison between measurements on the test bench and the finite elements simulation results will be shown.

## 2. Model Description

The macroscopic magnetization variation of ferromagnetic sheets results from the domain-walls displacement and the rotation of the magnetic moment occurring at a mesoscopic scale. These processes result from the competition of different energy terms well known at this scale: the exchange energy, the magnetocrystalline anisotropy energy, the magnetoelastic energy and the magnetostatic energy composed of the Zeeman and demagnetizing field energy.

In the presented model, which minimizes the macroscopic total energy with respect to the magnetization, the exchange energy term is not taken into account. In fact, the exchange interaction is an important energy term in ferromagnetic materials and explains the alignment of the magnetic moments of neighboring atoms inside domains. However this energy term is the same in all domains, so it doesn’t vary during the magnetization of sheets [10]. Therefore macroscopic magnetization depends on the domain volumes and the magnetization orientation inside domains.

Assuming an isothermal process, choosing the demagnetized and unstressed state as the reference state, the energy of a ferromagnetic volume at macroscopic scale can be decomposed [10,11] following Equation (1):

(1) $$E_{\mathrm{tot}}=E_{d}+E_{z}+E_{a}+E_{\sigma }$$

where:

- E_d_ is the demagnetizing field energy.
- E_z_ is the applied magnetic field (Zeeman) energy.
- E_a_ is the anisotropy energy.
- E_σ_ is the stress induced anisotropy.

The small thickness compared to the other dimensions of ferromagnetic sheet favors a magnetic flux parallel to the plane of the sheets. Therefore, a planar magnetization (**M**) is assumed, so it is convenient to use polar coordinates (k,θ) in Equation (2):

(2) $$\begin{array}{l} \left\|M\right\|=k\cdot M_{S}\left\{ \begin{array}{l} 0\leq k\leq 1\\ M_{S}\mathrm{Saturation}\;\mathrm{magnetization} \end{array}\right.\\ \left(M,RD\right)=\theta \left\{ \begin{array}{l} -\pi \leq \theta \leq \pi \\ \mathrm{RD}\mathrm{Rolling}\;\mathrm{direction} \end{array}\right. \end{array}$$

In the following section, the energy terms introduced in Equation (1) and already well detailed in our previous works will be presented [12].

## 3. Energy Terms Definition and Identification of Parameters

### 3.1. Demagnetizing Field Energy

The divergence of magnetic field is related to the divergence of magnetization (div **H** = −div **M**), creating a demagnetizing field **H_d_**= −N_d_·**M** in the ferromagnetic volume. The energy associated with this change of magnetic field is modeled in Equation (3):

(3) $$E_{d}=\frac{\mu_{0}}{2}N_{d}{\left\|M\right\|}^{2}$$

where:

- N_d_ is a factor to take into account the considered demagnetizing field.
- μ_0_ is the vacuum permeability.

In fact, the appearance of a demagnetizing field can be interpreted on different scales. On the one hand it can be induced by the geometry of the sample at a macroscopic scale. This geometry effect is included in the magnetic field analysis. On the other hand, some other local fields at the mesoscopic scale appear because of the differences in single crystal behavior. This effect is more important at the beginning of magnetization, because the differences of behavior are greater. In this model, which focuses on material behavior, only local demagnetizing fields are considered. Then the N_d_ factor is given by Equation (4) [12]:

(4) $$N_{d}=\frac{1}{\left(1-k^{2}\right)\chi_{i}\left(\theta_{H0}\right)}$$

**Figure 1:**
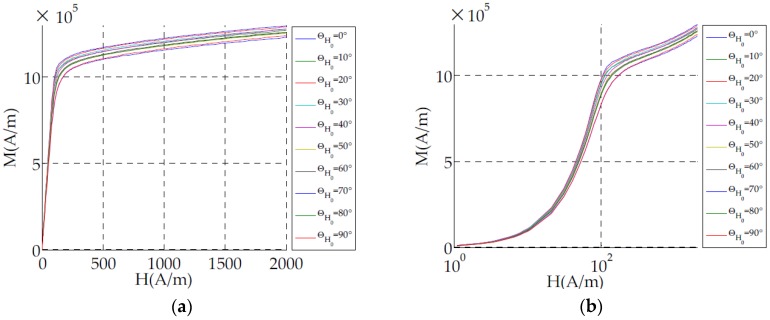
Non-hysteretic (anhysteretic) plotted magnetization curve according to the magnetic field directions: (**a**) linear; (**b**) logarithmic scale.

The parameter χ_i_(θ_H0_) can be measured from the linear part of the non-hysteretic (anhysteretic) plotted magnetization curves (Figure 1). In this figure, the magnetization of 10 sheets with the same shapes but cut in different directions is shown (Section 4.1). In Figure 1b the magnetization is plotted on a logarithmic scale to highlight the variation of the magnetic behavior at low magnetic field according to the cut directions.

### 3.2. Zeeman Energy

Equation (5) results from the interaction between the macroscopic magnetization and the external magnetic field:

(5) $$E_{z}=-\mu_{0}\cdot M\cdot H_{0}$$

### 3.3. Anisotropy Energy

**Figure 2:**
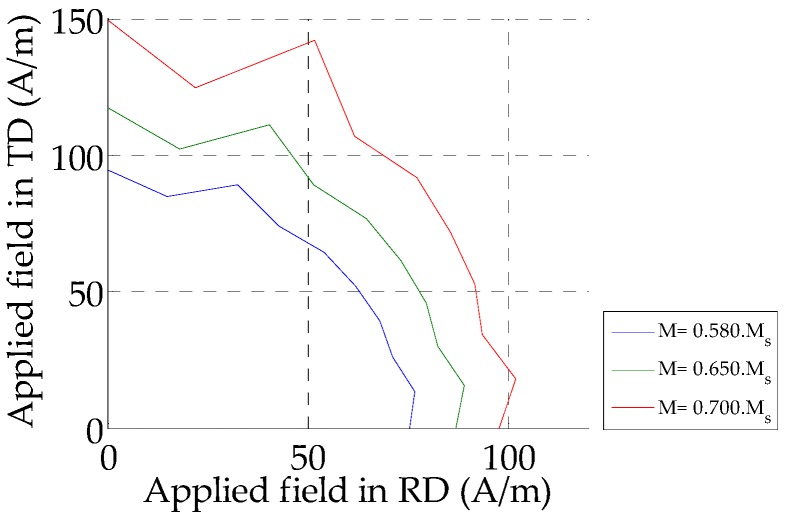
Polar representation of the magnetic field at three magnetization level of a NO SiFe sample.

Figure 2 below illustrates the magnitude of the magnetic field that must be applied in order to have the same magnetization in different directions (Figure 1). As shown in Figure 2, the laminated ferromagnetic polycrystal NO SiFe exhibits anisotropy at a macroscopic scale due to the material texture. It is well illustrated in [13] that sheet lamination affects the crystal orientations.

Considering the periodicity, the parity and the influence of the magnetocrystalline anisotropy on the rotation of magnetic moment, this energy term can be decomposed into a Fourier series multiplied by an exponential function (Equation (6)):

(6) $$E_{a}=\left(\exp\left(k^{\alpha }\right)-1\right)\cdot \left(\sum_{n=0}^{\infty }A_{n}\cdot \cos\left(n\theta \right)\right)\mathrm{With}\;n\;\mathrm{an}\;\mathrm{even}\;\mathrm{number}$$

This exponential function and the factor α are used to modulate the anisotropic effect which is crucial during magnetization rotation at high field. Since the anisotropy affects magnetization at high field, the parameters A_n_ are calculated near the saturation point thanks to the polar representation shown in Figure 2. In fact, assuming that the variation between two states of anhysteretic magnetization occurs without energy losses:

(7) $$\Delta E_{\mathrm{tot}}=E_{\mathrm{tot}\_f}-E_{\mathrm{tot}\_i}=0$$

E_tot_f_ and E_tot_i_ are respectively the total energy near the saturation and at reference chosen at a demagnetized state. Assuming that the demagnetizing field energy is negligible compared to the Zeeman energy and the applied magnetic field is parallel to magnetization at high field, then the anisotropic energy at the final state (E_a_f_) can be deduced from the Zeeman energy at the final state (E_z_f_) by Equation (8):

(8) $$E_{a\_f}\;\approx -E_{z\_f}$$

From relation Equation (8), the anisotropic energy according to the magnetization direction can be calculated experimentally (E_a_exp_(θ_exp_)) from the applied magnetic field **H_0_** using Equation (9):

(9) $$\left\{ \begin{array}{l} E_{a\_\exp}\approx \mu_{0}M_{s}\left\|H_{0}\right\|=\mu_{0}M_{s}\sqrt{H_{0\_\mathrm{RDexp}}^{2}+H_{0\_\mathrm{TDexp}}^{2}}\\ \theta_{\exp}\approx \arctan\left(\frac{H_{0\_\mathrm{TDexp}}}{H_{0\_\mathrm{RDexp}}}\right) \end{array}\right.$$

Then, the parameters A_n_ can be deduced from the experimental applied magnetic field by calculating the Fourier coefficients:

(10) $$\left\{ \begin{array}{l} A_{0}=\langle E_{a\_\mathrm{expj}}\rangle =\frac{1}{N}\sum_{j=0}^{N-1}E_{a\_\mathrm{expj}}\\ A_{n}=\frac{2}{N}\sum_{j=0}^{N-1}E_{a\_\mathrm{expj}}\cdot \cos(n\cdot \theta_{\mathrm{expj}}) \end{array}\right.$$

N and θ_expj_ are the number and the angles of measuring directions.

### 3.4. Stress-Induced Anisotropy

The domain structure of ferromagnetic materials is sensitive to stress. Then, the application of an external stress induces a variation of the macroscopic magnetization. This phenomenon is due to the magnetoelastic coupling and is modeled by the energy term given by Equation (11):

(11) $$E_{\sigma }=-\underline{\underline{\sigma }}:{\underline{\underline{S}}}^{\mu }$$

where σ and S^μ^ are respectively the stress and the magnetostriction tensors given in the material coordinate system (RD,TD).

Since the anisotropic magnetostriction, which varies during the use of ferromagnetic materials, is isochoric, it can be written in the magnetization coordinate system by:

(12) $${\underline{\underline{S}}}_{\vec{M}}^{\mu }=\left(\lambda_{A}\left(\theta \right)\cdot k^{2}+\lambda_{B}\left(\theta \right)\cdot k^{4}+...\right)\left(\begin{array}{l} \begin{array}{cc} 1 & 0 \end{array}\\ \begin{array}{cc} 0 & \frac{-1}{2} \end{array} \end{array}\right)$$

The rotation matrix **P** is used to write magnetostriction in the material coordinate system (RD,TD):

(13) $$\underline{\underline{\sigma }}=\left(\begin{array}{l} \begin{array}{cc} \sigma_{\mathrm{RD}} & \sigma_{\mathrm{RTD}} \end{array}\\ \begin{array}{cc} \sigma_{\mathrm{RTD}} & \sigma_{\mathrm{TD}} \end{array} \end{array}\right);\;{\underline{\underline{S}}}^{\mu }=P\cdot {\underline{\underline{S}}}_{\vec{M}}^{\mu }\cdot P^{-1};\;P=\left(\begin{array}{l} \begin{array}{cc} \cos(\theta ) & -\sin(\theta ) \end{array}\\ \begin{array}{cc} \sin(\theta ) & \cos(\theta ) \end{array} \end{array}\right)$$

The magnetostriction strain is anisotropic and depends on the magnetization orientation [14]. Therefore functions λ_A_(θ), λ_B_(θ) are identified thanks to magnetostriction measurements near the saturation point in different directions. In fact, according to the magnetostriction tensor (Equation (12)), the measured magnetostriciton in a given direction β is:

(14) $$\lambda_{\beta }^{\mu }={\frac{\Delta l}{l}|}_{\beta }=\sum_{i,j}s_{\mathrm{ij}}^{\mu }\beta_{i}\beta_{j}$$

Assuming that near the saturation β ≈ θ, then the measured deformation becomes:

(15) $$\lambda_{\beta }^{\mu }={\frac{\Delta l}{l}|}_{\beta \approx \theta }=\lambda_{A}\left(\theta \right)\cdot k^{2}+\lambda_{B}\left(\theta \right)\cdot k^{4}+...$$

## 4. Validation of the Model and Integration in a Finite Elements Simulation

Since the parameters of the energy terms are identified from measurements (Equations (4), (10) and (15)), the magnetization and the magnetostriction can be computed by minimizing the total energy (Equation (1)) with respect to k and θ. The global minimum of the total energy function corresponds to the anhysteretic state.

### 4.1. Comparison between Experiments on Single Sheet and Calculated Values

**Figure 3:**
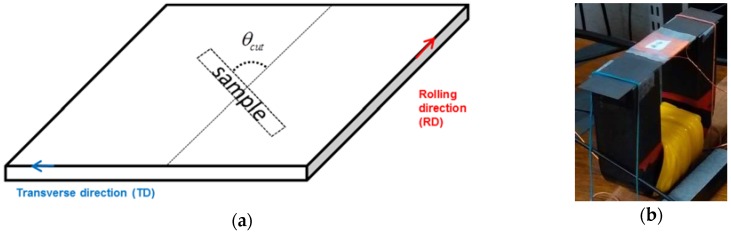
Single Sheet experimental procedure: (**a**) sampling; (**b**) sample’s magnetization circuit.

This model is already used to predict magnetostriction and magnetization measurement from [12]. In order to validate the model, the anhysteretic magnetization and magnetostriction of a non-oriented silicon sheet (Fe-3%Si) are measured along 10 directions (from the rolling to the transverse directions) thanks to samples cut in the corresponding angles (Figure 3a). All samples have the same size (150 mm long, 30 mm wide and 0.35 mm thick) and are magnetized at low frequency (1.5 Hz) via a coil fed by a current generator and wound around a U-shape core (Figure 3b).

The magnetization is deduced from the flux density and the magnetic field respectively measured by a b-coil and a h-coil (Equation (16)). Therefore:

(16) $$M=\frac{B}{\mu_{0}}-H$$

where:

- M is the magnetization.
- B is the flux density measured by a b-coil (Figure 3b)
- μ_0_ is the vacuum permeability.
- H is the magnetic field measured by a H-coil.

The magnetostriction is measured by a strain gauge which the factor is 1.93 and the resistance is 350 Ω. The strain gauge is connected to a Wheatstone bridge. Then the output of the Wheatstone bridge is filtered thanks to a low-pass filter (cutoff frequency of 300 Hz) to attenuate high frequency measurement noises. Then the filtered signal is amplified thanks to a voltage amplifier (gain amplifier 25) to improve the resolution of the acquired magnetostriction signal.

**Figure 4:**
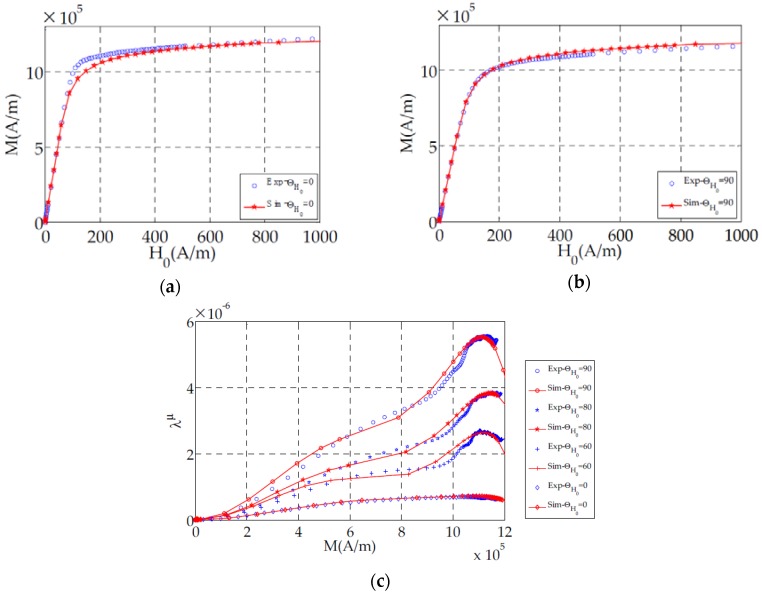
(**a,b**) Non-hysteretic (anhysteretic) plotted magnetization curves along RD (0°) and TD (90°); (**c**) Longitudinal magnetostriction in different magnetic field directions.

As illustrated in Figure 4, there is a good agreement between model simulations and single sheet measurements in different magnetic field directions. Then, this model is interesting for electric device applications. Since both multidirectional magnetostriction and magnetization are predicted, simulation of ferromagnetic sheets excited by rotational magnetic fields can be more accurate.

### 4.2. Integration in a Finite Elements Simulation and Comparison between Simulation and Measurements

**Figure 5:**
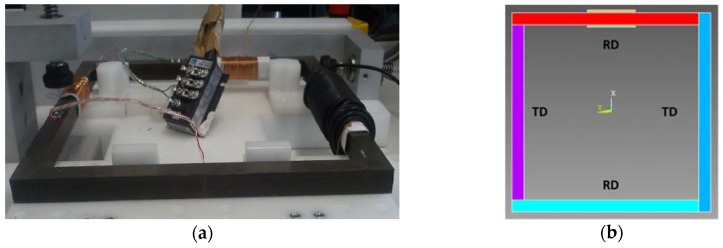
In house test bench: (**a**) real; (**b**) modeled.

In this section, the previously presented model is used to predict the magnetostrictive deformation of an in-house ferromagnetic frame Figure 5. In order to measure only magnetostriction effects, the ferromagnetic core is composed by a stacking of 40 closed sheets. These sheets are impregnated like transformers. Therefore, the magnetic circuit doesn’t include an air gap which could induce other magnetic forces. An 80 turn coil, fed by an autotransformer, creates a magnetic field through the frame. The outer perimeter measures 1000 mm (4 × 250 mm) and each column is 15 mm wide and roughly (40 × 0.35 mm) thick.

Concerning the simulation (Figure 5b), the columns are modeled as four materials (two RD and two TD) to take into account the difference between magnetic and magnetostrictive behavior.

**Figure 6:**
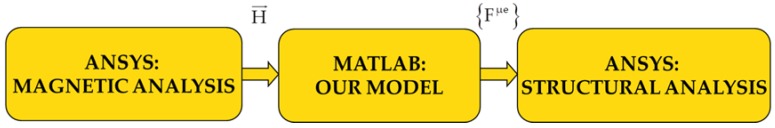
Forward coupling used to compute the magnetostriction strain of the test bench.

Because of the mechanical stresses induced by magnetostriction in the case of this material are too low (less than 1 MPa) to affect the magnetization state, a forward coupling (Figure 6) will be used to simulate the magnetostriction of the frame.

**Figure 7:**
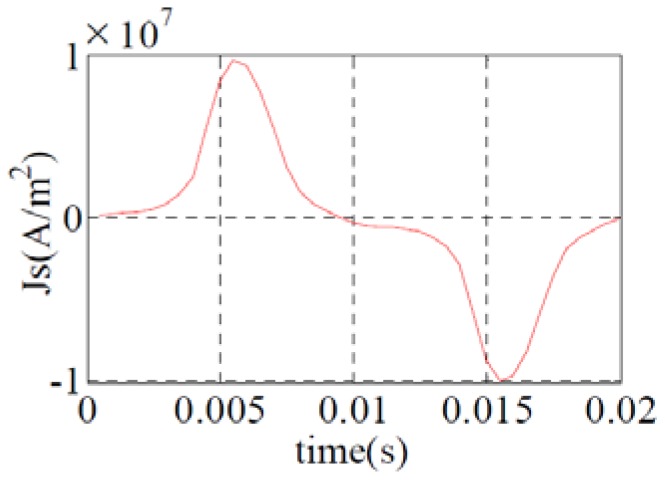
Measured current density: 50 Hz.

Then, in a first phase, ANSYS software is used to compute the magnetic field distribution according to the applied coil current (Figure 7).

The magnetic field distribution result is used in our model to calculate the magnetostriction of each element. Because magnetostriction is considered to be elastic strain, equivalent nodal forces are calculated thanks to Hooke’s law which depends on Young modulus and Poisson coefficient parameters (Equation (17)):

(17) $$\begin{array}{l} \left\{F^{\mu e}\right\}={\int_{v_{e}}\left[B\right]}^{T}\left\{\sigma^{\mu }\right\}dv\\ \sigma^{\mu }=C\left(E,\nu \right).S^{\mu } \end{array}$$

where :

- {F^μe^} is the nodal forces vector of an element.
- v_e_ is the element volume.
- [B] is the matrix of shape function depending on the chosen element.
- {**σ**^μ^} is the equivalent magnetostriction stress in Voigt notation.
- C(E,ν) is the stiffness matrix depends on the Young modulus (E) and Poisson ratio (ν).
- **S**^μ^ is the magnetostriction strain in Voigt notation.

**Figure 8:**
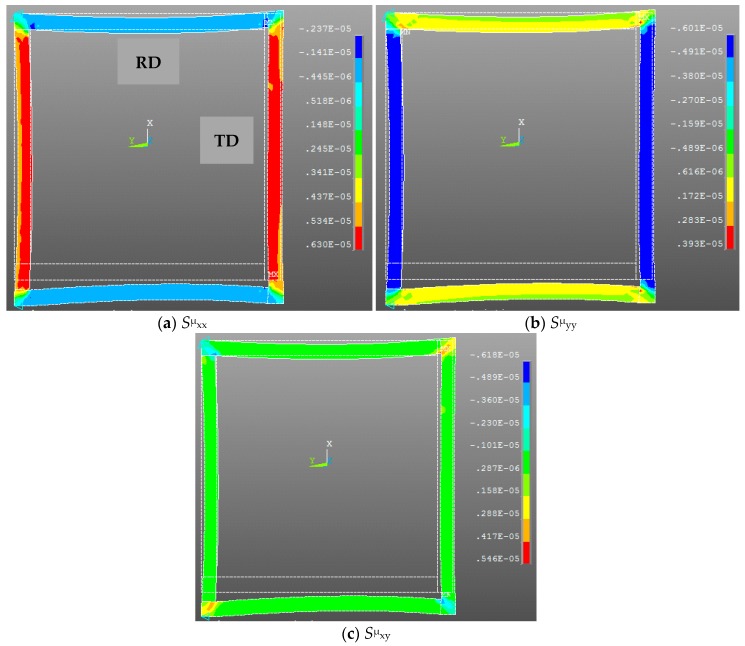
Simulated magnetostriction of the test bench (at the same point corresponding to the maximum strain along the TD column): Normal magnetostrictive strain along the X-axis (**a**); Normal magnetostrictive strain along the Y-axis (**b**); Shear magnetostrictive strain XY (**c**).

Finally all nodal forces are applied to the ferromagnetic core in an ANSYS mechanical analysis environment after assembling the element nodal forces (Figure 6). To avoid rigid body mode (rotation of the frame around the Z axis and Y displacements), boundary conditions are applied at two nodes at the corners (Figure 8).

Since the magnetostriction is an even function and its frequency is twice the frequency of the applied magnetic field (or the applied current shown in Figure 7), the simulation is done for a quarter of the current period (between 0 to 5 ms).

In Figure 8 normal magnetostriction strains (S^μ^_xx_ and S^μ^_yy_) and shear magnetostriction strain (S^μ^_xy_) are mapped on the deformed frame in the global coordinates system (X,Y). As expected and illustrated on Figure 4, magnetostriction is roughly six times higher along TD (Figure 8a) than RD (Figure 8b). We can also notice that the model allowed us to predict shear magnetostriction which can affect the simulation results depending on the geometry of the studied frame. In fact, as we can observe in Figure 8c that this magnetostriction component is linked to the flux rotation which is particularly important at the corner of the frame.

**Figure 9:**
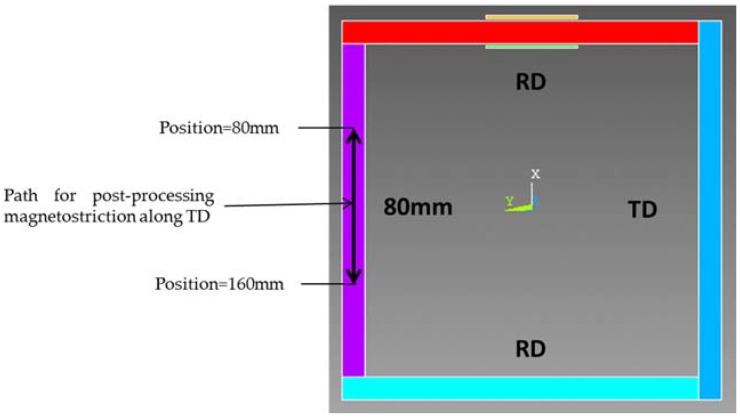
Computing magnetostriction along TD.

Since magnetostriction along the TD direction is more important than RD, our study was focused on the TD direction. Then a strain gage is glued at the middle of TD column (Figure 5) to measure the strain. On the other hand, magnetostriction is computed along a path defined inside the TD column 40 mm on both sides of the middle (Figure 9).

**Figure 10:**
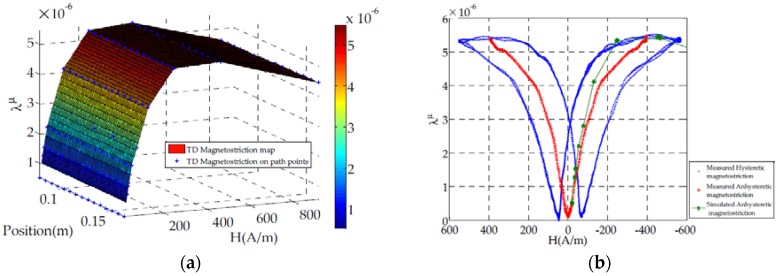
Comparison between measured and simulated magnetostriction along the TD column. (**a**) on path (Figure 9); (**b**) at TD-column middle (Figure 9).

The averaged magnetostriction along the TD column width is plotted in Figure 10a at different positions on the defined path. We can notice that TD column magnetostriction doesn’t vary according the position (Figure 10a). Therefore the computed magnetostriction at the middle of the TD column is compared to the strain guage measurement.

The magnetostriction is measured for several periods and represented as a function of magnetic field. This representation shows a hysteresis loop due to the well-known hysteresis phenomenon in magnetic behavior (Figure 10b). Because of our model is anhysteretic, the simulated magnetostriction is compared to the mean curve from the measured hysteretic magnetostriction (Figure 10b).

## 5. Conclusions

A macroscopic model based on energy minimization is proposed. This model takes into account anisotropy effects in both magnetization and magnetostriction computation thanks to different energy terms. These energy terms, derived from experimental results, attempt to give physical sense to macroscopic scale measurements.

The model shows a good agreement with the multidirectional magnetization and magnetostriction measurement on a single sheet tester (Figure 4). Therefore it would be interesting to use it in applications such as electric motors, magnetostrictive actuators, *etc.* A finite element method based on calculating magnetostriction nodal forces is used to apply our model to a test bench which has a similar topology to a transformer. According to Figure 10b, a good agreement is obtained for the TD direction which magnetostriction is six times higher than in the RD direction. However we can notice (Figure 10b) that at the technical saturation (around H ≃ 200 A/m), the error between measured and simulated magnetostriction reaches a maximum value. This can be due to the saturation of the material. In fact, saturated material implies an increasing magnetic flux leakage which can generate magnetic forces on the test bench surface (for example Maxwell forces).

Concerning measurement, in our future work, a laser vibrometer will be used to measure the test bench surface velocity for comparison with the simulation results. Flux leakage will be also measured to add potential magnetic forces in the model. Concerning the model, since anhysteretic magnetization and magnetostriction are well estimated, hysteretic behavior can be deduced. Therefore in the future, frequency effectss will be added to the model. Including frequency effects in magnetostriction computing can be interesting for transformer and electric motors connected to power electronic converters.

## Abbreviations

The following abbreviations are used in this manuscript:

VPI	Vacuum-Pressure Impregnation
RD	Rolling Direction
TD	Transverse Direction
